# Investigating students’ intention to utilize an e-learning platform in an aviation institution during the COVID-19 pandemic

**DOI:** 10.1371/journal.pone.0308180

**Published:** 2024-12-31

**Authors:** Anthony Vicente M. Belo, Yogi Tri Prasetyo, Ralph Andre C. Roque, Omar Paolo Benito, Maela Madel L. Cahigas, Ma. Janice J. Gumasing, Rianina D. Borres, Reny Nadlifatin

**Affiliations:** 1 School of Industrial Engineering and Engineering & Management, Mapua University, Manila, Philippines; 2 School of Graduate Studies, Mapua University, Manila, Philippines; 3 International Bachelor Program in Engineering, Yuan Ze University, Chung-Li, Taiwan; 4 Department of Industrial Engineering and Management, Yuan Ze University, Chung-Li, Taiwan; 5 Department of Industrial and Systems Engineering, Gokongwei College of Engineering, De La Salle University, Manila, Philippines; 6 Department of Information Systems, Institut Teknologi Sepuluh Nopember, Kampus ITS Sukolilo, Surabaya, Indonesia; Al-Ahliyya Amman University, JORDAN

## Abstract

Aviation College is a higher education institution that shifted to e-Learning as the education platform during the COVID-19 Pandemic. This shift has posed challenges, especially in developing countries like the Philippines. This study aims to evaluate students’ intentions toward using an e-learning platform at a collegiate aviation institution during the pandemic by employing an integrated extended Technology Acceptance Model (TAM) and Seddon’s Information System (IS) Success Model. The study involved 503 college students who completed an online questionnaire with 48 items representing 12 constructs. Structural Equation Modeling (SEM) was utilized to analyze the relationships between variables under extended TAM and IS Success Model. The findings revealed that attitude toward use had the strongest influence on behavioral intention, followed by perceived playfulness. Learning outcomes significantly impacted perceived usefulness, along with information quality, perceived ease of use, and system quality. Additionally, learning outcomes had the greatest effect on user satisfaction, followed by perceived usefulness, information quality, and system quality. Perceived usefulness had a more substantial impact on attitude toward use than perceived ease of use. Regarding perceived ease of use, system quality was the most influential factor, followed by computer self-efficacy and course design. The proposed framework enhances understanding of the relationship between technology adoption theory and the IS success model. The study’s findings can help policymakers, software developers, and educators improve the e-learning process and maintain the quality of education.

## 1. Introduction

E-learning has been the essential mode of instruction due to the ongoing COVID-19 pandemic restrictions. This health crisis has affected more than 1.5 billion students [1, 2]. To prevent disruption in education, e-learning platforms such as Google Classroom, Blackboard, and Moodle have facilitated distance learning and social interaction. However, educational institutions are challenged to implement a remote learning system that meets their student’s needs [3]. Thus, understanding success factors and learner acceptance is key to successfully enforcing e-learning.

Aviation colleges in the Philippines provide flight training, aircraft maintenance, aeronautical engineering, and airline management courses. While flight training cannot be conducted remotely, other courses such as aviation management or airport planning can still be online [4]. In an accredited aviation college in the Philippines, an average of 83% of the courses offered in the Bachelor of Science programs in Aeronautical Engineering, Aircraft Maintenance Technology, Air Transportation, and Avionics Technology can be conducted online. However, several studies have emphasized that the attitudes and perceptions of students directly influence their motivation, engagement, and eventually, the overall effectiveness of remote learning [5–7]. In addition, the International Civil Aviation Organization (ICAO) [8] has projected a future shortage of aviation professionals, reinforcing the importance of the perception and engagement of students with technology, and the overall learning experience in the success of remote learning [9, 10]. Given that aviation institutions are at the forefront of educating future professionals, providing quality education to aviation students through remote learning is crucial.

This study proposes an integrated extended TAM and Seddon’s IS Success Model to evaluate the students’ Intentions toward an e-learning platform in a collegiate aviation institution in the Philippines during the COVID-19 Pandemic. The proposed research model will also be used to determine the relationship between constructs and to build an understanding of how these models can complement each other [11]. This paper is the first to explore the gap in e-learning studies in the Philippines in the context of collegiate aviation amidst the ongoing global health crisis. This research could support policymakers and educators in collegiate aviation in assessing the successful implementation of their e-learning infrastructure. Moreover, this study may contribute to formulating policies grounded on the empirical understanding of e-learning acceptance in Higher Education in the Philippines and support its sustained development [12].

## 2. Literature review and hypothesis development

The technology acceptance model (TAM) introduced by Davis is widely used to understand the adoption of information systems or technology [13]. Several studies have tried to understand e-learning acceptance and determine its success factors to maximize effectiveness using TAM. A study in the United Arab Emirates used TAM to evaluate the Intention of e-learning in five universities [14]. Computer playfulness, system quality, and self-efficacy affected perceived ease of use. At the same time, perceived ease of use and Usefulness were affected by accessibility, information quality, and perceived enjoyment [14]. In Korea, the study by Han and Sa [1] found that user satisfaction was positively influenced by usefulness and ease of use. Another study by Lee et al. [3] added playfulness, design of learning contents, teaching materials, and instructor characteristics to the TAM model. Meanwhile, a systematic literature review conducted by Meet and Kala [15] gathered one hundred and two (102) previous literature during the period 2013–2023 that were related to technology adoption in the education sector. They revealed that the most widely used and accepted theoretical model in the sampled literature is the TAM (18.03%). It was also used alone in eleven (11) papers and another five (5) as an integrated model. They also stated that the reason for this is many of the papers focus on the theme of Massive Open Online Course (MOOC) adoption. This means researchers are often investigating how and why people accept and use MOOCs [16].

Several studies that integrated TAM with other theories to further understand e-learning acceptance also presented plenty of meaningful results. A study by Al-Adwan et al. [17] integrated TAM with the theory of Technological Pedagogical Content Knowledge (TPAK) and the Unified Theory of Acceptance and use of Technology (UTAUT) to determine the continuous use intention of technology among teachers in higher education institutions (HEI). They highlighted that perceived usefulness, perceived ease of use, self-efficacy, and social influence have a major influence on their continuous use intention of technology. They also noted that their integrated model established an explanatory power of 60.4%. Similarly, Meet et al. [18] aimed to determine the factors that influence the behavioral intention among Generation Z in India to adopt the use of MOOCs. They highlighted the significant impact of effort expectancy, performance expectancy, hedonic motivation, facilitating conditions, and price value on MOOC adoption. In addition, Davis [19], mentioned that when external variables are included in TAM, it should be specific to the technology being evaluated [20]. Based on the successful findings of the previously mentioned research studies, researchers have not only incorporated external variables but also integrated TAM with several other theories. One of the theories that can be integrated with TAM to investigate the successful implementation of information systems is the Information Systems (IS) Success Model.

The Information System (IS) Success Model by DeLone and McLean (D&M) has been employed in various settings to explain system use, user satisfaction, and IS success [21–23]. However, Seddon respecified his model due to the lack of empirical support and recommendation [24]. According to Seddon [24], his model provides more precise and theoretically sound connections between constructs. The study by Rai et al. [25] assessed DeLone and McLean’s [23] model and Seddon’s [24] model. Rai et al. [25] stated that the Seddon model elaborates on the causal structure of TAM and parallels the specifications of TAM and the Theory of Planned Behavior (TPB). The study of Gonzales and Wareham [26] assessed the D&M’s model, the Seddon model, and the Modified Seddon model. Findings showed that Seddon’s Model is the best fit. Therefore, utilizing these frameworks could further the study’s objective of rationalizing adopting information systems such as e-learning.

Understanding e-learning acceptance and determining its success factors will benefit the rapid transition from traditional to online learning. Most schools, colleges, and universities still do not use this method before the COVID-19 pandemic [27]. In developing countries such as the Philippines, the use of such technology is still in its early stages [28, 29]. The country’s Commission on Higher Education (CHED) still does not have a national policy on distance learning or e-learning [30]. Because of this, Higher Educational Institutions (HEIs), including aviation colleges, were left to formulate their policies for remote online learning.

**Fig 1:**
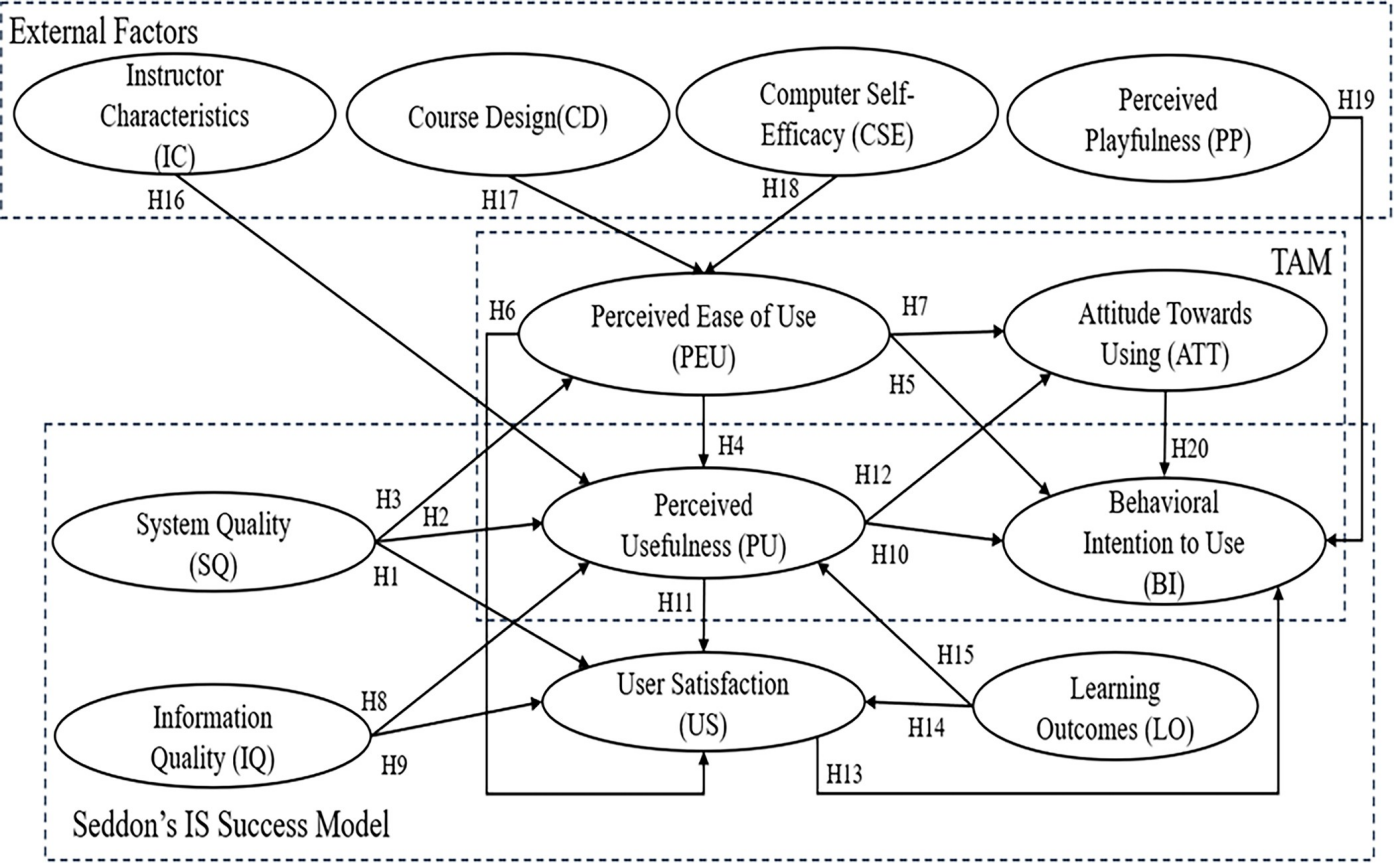
Research framework.

Fig 1 presents the research framework of the study. It is an integration of the extended TAM and Seddon’s IS Success Model, which includes external factors, namely Instructor Characteristics (IC), Course Design (CD), Computer Self-efficacy (CSE), and Perceived Playfulness (PP).

System Quality (SQ) defines the usability, reliability, availability, and adaptability of the use of an e-learning system [14]. An increase in system quality increases user satisfaction and the perceived usefulness of a system [24, 31, 32]. In addition, the findings of Salloum et al. [14] and Prasetyo et al. [33] found that system quality affects perceived ease of use. From these, the researchers hypothesize that:

***H1*:** *System Quality has a significant effect on User Satisfaction*.
***H2*:** *System Quality has a significant effect on Perceived Usefulness*.
***H3*:** *System Quality has a significant effect on Perceived Ease of Use*.

Perceived Ease of Use (PEU) is how a user believes using the technology would be effortless [19]. Using a system free of effort is more probable to be accepted by the user [19]. Empirical evidence has implied that PEU is directly and indirectly linked to behavioral Intention through perceived usefulness [19, 34–36]. A study by Rai et al. [25] also highlighted that PEU positively impacts Usefulness and satisfaction. In addition, previous online learning acceptance research has demonstrated that ease of use influences attitude toward using [14, 37–39]. Thus, the researchers hypothesize the following:

***H4*:** *Perceived Ease of Use has a significant effect on Perceived Usefulness*.
***H5*:** *Perceived Ease of Use has a significant effect on Behavioral Intention to Use*.
***H6*:** *Perceived Ease of Use has a significant effect on User Satisfaction*.
***H7*:** *Perceived Ease of Use has a significant effect on Attitude Towards Using*.

Information Quality (IQ) pertains to the degree the user of e-learning can seek relevant, timely, and accurate information [14, 24]. IQ is an essential factor in assessing the success of e-learning because it is crucial in achieving learning goals [40, 41]. Previous studies support that IQ affects Usefulness and user satisfaction [25, 31, 40, 42, 43]. From this, the researchers hypothesized that:

***H8*:** *Information Quality has a significant effect on Perceived Usefulness*.
***H9*:** *Information Quality has a significant effect on User Satisfaction*.

Perceived Usefulness (PU) is a perceptual measure of the extent to which the user considers that using a system will improve his or her performance and produce benefits [24]. Seddon and Kiew’s [31] empirical work stated that each user has expectations for the system that must be met. Therefore, the more valuable the tool, the more likely the user is satisfied. In e-learning, PU significantly affects behavioral intent based on previous studies [3, 36, 44–46]. This construct has also been empirically proven to influence attitudes towards use [14, 37–39]. Thus, the researchers hypothesize the following:

***H10*:** *Perceived Usefulness has a significant effect on Behavioral Intention to Use*.
***H11*:** *Perceived Usefulness has a significant effect on User Satisfaction*.
***H12*:** *Perceived Usefulness has a significant effect on Attitude Towards Using*.

User Satisfaction (US) is the overall feeling after using the technology [44]. Similarly, the study by Hassanzadeh et al. [47] stated that when users are satisfied, their loyalty to using the platform increases. Thus, the researchers hypothesize that:

***H13*:** *User Satisfaction has a significant effect on Behavioral Intention to Use*.

Learning Outcomes (LO) refer to the perceived individual net benefit derived from using the e-learning tool [44]. In this study, the construct focuses on fostering knowledge-building, improving the learning process, and achieving goals [40]. The model is intended to be consistent with Seddon [24] and Hsieh and Cho [44], wherein "other measures of net benefits" such as LO affect the US and PU. Thus, the researchers hypothesized that:

***H14*:** *Learning Outcomes has a significant effect on User Satisfaction*.
***H15*:** *Learning Outcomes has a significant effect on Perceived Usefulness*.

Instructor Characteristics (IC) are the degree to which instructors support their students [3]. The instructor’s attitude toward e-learning and pedagogy affects the learner’s participation and attitude toward e-learning [3, 40, 45, 48]. Past studies indicate that IC affects PU [3, 40, 42]. Thus, the researchers hypothesize that:

***H16*:** *Instructor Characteristics has a significant effect on Perceived Usefulness*.

Course Design (CD) is the extent to which learning contents are structured and developed to suit students’ needs [3]. Sun et al. [45] stated that course content should be carefully designed for a better learning experience. Lee et al. [3], found that CD was an essential indicator of PEU. Thus, the researchers hypothesize that:

***H17*:** *Course Design has a significant effect on Perceived Ease of Use*.

Computer Self-Efficacy (CSE) measures users’ confidence in using computer systems in their capacity [14]. The extensive review of Salloum et al. [14] has shown that CSE is TAM’s most widely utilized external construct. Previous studies have shown that CSE significantly affects PEU [14, 49]. Thus, the researchers hypothesize that:

***H18*:** *Computer Self-Efficacy has a significant effect on Perceived Ease of Use*.

Perceived Playfulness (PP) is a hedonic outcome such as pleasure, enjoyment, or happiness derived from the use of technology [50]. It pertains to the intrinsic motivational factor of using a new system [14, 50]. Lee et al. [3] concluded that PP affects the Intention to use e-learning. Hence, the researchers hypothesized that:

***H19*:** *Perceived Playfulness has a significant effect on Behavioral Intention to Use*.

Behavioral Intention to Use (BI) refers to the learner’s desire to use e-learning from the present to the future [14]. This construct evaluates the degree of the user’s commitment to engage in the specified behavior [51].

Attitudes are the predisposition of human behavior from a consistently favorable or unfavorable reaction to an object [37, 52, 53]. In this study, attitude characterizes the positive or negative feelings toward e-learning [14, 54]. Previous studies have empirically proven that attitude directly affects behavioral intention [14, 37–39, 54, 55]. Thus, the researchers hypothesize that:

***H20*:** *Attitude Towards Use has a significant effect on Behavioral Intention to Use*.

## 3. Methods

### 3.1. Structural Equation Modeling

Structural Equation Modeling (SEM) is a multivariate analysis method that allows the researcher to examine the relationships between variables and latent constructs [56]. The interrelationships between constructs are linked based on the hypotheses. This study utilized SmartPLS 3 to run the structural equation model. Indicators used to measure the adequacy of model fit were the following: Standardized Root Mean Square (SRMR) of 0.00 to 0.08 [57] and Normed Fit Index (NFI) of 0.60 to 1.00 [58].

### 3.2. Participants

This research employed non-probability purposive sampling targeting a particular population category [59]. The 503 participants were users of the e-learning platform at an accredited aviation educational institution in the Philippines and the data were collected from July 1^st^, 2021 to December 1^st^, 2021. Due to the COVID-19 pandemic, the questionnaire was distributed in a 3-week time frame through social media channels via a sent link to an online survey form created in Google Forms. This study was approved by Mapua University Research Ethics Committees (FM-RC-21-49) and each respondent agreed to participate by signing an online consent form. Prior to the data collection, each of them was informed of their responses’ confidentiality.

### 3.3. Questionnaire

The questionnaire consisted of two parts. The first part was composed of (6) demographic profile questions, namely gender, age, program taken, year level, time spent using the platform per week, and usage frequency per week. The second section had 48 items or indicators for the twelve (12) latent variables (Table 1). These indicator statements were gathered from previous literature related to technology adoption in the education sector. The level of agreement of each respondent was measured using the 5-point Likert scale. It consists of five points (1) Strongly Disagree, (2) Disagree, (3) Neither Agree nor Disagree, (4) Agree, and (5) Strongly Agree [60]. In addition, a pilot test was conducted to assess the reliability and validity of the questionnaire.

**Table 1:** Questionnaire.

Construct	Item	Measure	Supporting References
System Quality (SQ)	SQ1	Google Classroom’s interface and system design is friendly.	[14, 33]
	SQ2	I feel comfortable using Google Classroom’s services and functionalities.	
	SQ3	I consider Google Classroom’s functions to be satisfactory.	
	SQ4	I consider the interaction with Google Classroom to be satisfactory.	
Perceived Ease of Use (PEU)	PEU1	There is clarity and understanding in my interaction with Google Classroom.	[1]
	PEU2	Google Classroom is easy to use for me.	
	PEU3	Interacting with the Google Classroom system does not require a lot of mental effort.	
	PEU4	My interaction with the Google Classroom is clear and understandable.	
Information Quality	IQ1	Google Classroom provides sufficient information for my study.	[40, 41]
	IQ2	Google Classroom provides accurate information for my study.	
	IQ3	Google Classroom provides useful information for my study.	
	IQ4	Google Classroom provides relevant information for my study.	
Perceived Usefulness	PU1	The use of Google Classroom enabled me to accomplish certain tasks more quickly.	[14]
	PU2	The use of Google Classroom improved the quality of my tasks.	
	PU3	The use of Google Classroom enhanced the effectiveness of my tasks.	
	PU4	Google Classroom is useful to me.	
User Satisfaction	US1	Using Google Classroom would give me a better opportunity to explore the subject.	[44, 47]
	US2	I am satisfied with the performance of Google Classroom.	
	US3	Google Classroom satisfies my educational needs.	
	US4	Using Google Classroom gave me a sense of satisfaction.	
Learning Outcomes	LO1	Google Classroom improves my grade on the subject.	[40]
	LO2	Using Google Classroom has increased my knowledge and helped me to be successful in the module.	
	LO3	Google Classroom is a very effective educational tool and has helped me improve my learning process.	
	LO4	Google Classroom has helped me achieve the learning goals of the module.	
Instructor Characteristics	IC1	The instructor provides high-quality instruction.	[2]
	IC2	The instructor provides information on learning progress.	
	IC3	The instructor delivers instructions clearly.	
	IC4	Overall, the instructor’s attitude is conducive to learners’ learning via the e-learning system.	
Course Design	CD1	The level of difficulty of the learning contents is appropriate.	[3]
	CD2	The course materials were placed online on time.	
	CD3	The delivery schedule of learning content is flexible.	
	CD4	Google Classroom provides a variety of learning methods.	
Computer Self-Efficacy	CSE1	I feel confident in the utilization of Google Classroom even when no one is there for assistance.	[14]
	CSE2	I have sufficient skills to use Google Classroom.	
	CSE3	I feel confident when using Google Classroom even if I have only the online instructions.	
	CSE4	I feel confident when using the online learning content in Google Classroom.	
Perceived Playfulness	PP1	I feel Google Classroom helps me improve my creativity.	[3, 50]
	PP2	I feel Google Classroom helps me improve my imagination by obtaining information.	
	PP3	I feel I can have a variety of experiences without any interference.	
	PP4	I feel Google Classroom is fun regardless of usage purposes.	
Behavioral Intention to Use	BI1	I prefer using Google Classroom to traditional learning.	[14, 39, 55]
	BI2	I will recommend Google Classroom to other students.	
	BI3	I will use Google Classroom regularly in the future.	
	BI4	I intend to make use of the content and functions of Google Classroom to assist with my academic activities.	
Attitude Towards Using	ATT1	I feel positive regarding the utilization of Google Classroom.	[37, 38]
	ATT2	In general, I admire the utilization of Google Classroom.	
	ATT3	Google Classroom provides an attractive learning environment.	
	ATT4	Overall, I like using Google Classroom.	

## 4. Data analysis and findings

Table 2 exhibits the descriptive statistics of the respondents. The majority of the respondents were at the age of 18 to 24 years old (mean: 21.02 years; sd: 2.58 years) and mostly male (77.14%). 298 participants were taking up a Bachelor of Science in Aeronautical Engineering. For the time spent using the eLearning platform, the respondent population mainly utilizes it more than 4 hours per week. The majority (60.24%) use the platform daily.

**Table 2:** Descriptive statistics of respondents (n = 503).

Measure	Value	n	%
Gender	Male	388	77.14
	Female	115	22.86
Age	18 to 24 years old	497	98.81
	25 to 34 years old	6	1.19
Program/Course being taken	BS Aeronautical Engineering	298	59.24
	BS Air Transportation	128	25.45
	BS Aircraft Maintenance Technology	13	2.58
	BS Avionics Technology	36	7.16
	Pre-Engineering	28	5.57
Year Level	1st Year	55	10.93
	2nd Year	106	21.07
	3rd Year	255	50.70
	4th Year	85	16.90
	5th Year	1	0.20
	Irregular	1	0.20
Time Spent using e-learning platform per week	Less than 1 hour	3	0.60
	1–2 hours	10	1.99
	3–4 hours	44	8.75
	More than 4 hours	446	88.67
Usage Frequency of e-learning platform per week	1–2 times	11	2.19
	3–6 times	124	24.65
	7–12 times	65	12.92
	Daily	303	60.24

### 4.1. Measurement model

The partial least squares (PLS) method was used to analyze the data gathered from the respondents. It has been highlighted by Meet et al. [18] that PLS-SEM is adequate and considered to be accurate for explanatory power validation and appraising complex models [18]. In addition, it can also examine both the structural model and measurement model simultaneously [18]. Thus, the SmartPLS3 software was employed to run the PLS-SEM.

Table 3 presents the mean, standard deviation, standardized loadings, average variance extracted (AVE), composite reliability (CR), and Cronbach’s α. The standard deviation (SD) of indicators fell below +2. The highest SD is BI1, with a value of 1.431. Standardized loadings on all indicators were more outstanding than 0.5, which is significant and confirms indicator reliability [56]. Thus, all of the indicators were representative of their respective latent variables. According to Hair et al. [56], an AVE value higher than 0.5 implies a close relation of indicators to the latent construct and validates convergent validity. Results showed that all values were higher than 0.5. For CR, a benchmark value of 0.7 signifies internal consistency reliability. For Cronbach’s α, all values on each latent variable were higher than 0.7, suggesting the consistency and reliability of the measures used [56]. With these results, the indicator reliability, internal consistency reliability, and convergent validity is established.

**Table 3:** Construct validity and reliability.

Latent Variable	Indicator	Loadings	Mean	Standard Deviation	VIF	AVE	CR	Cronbach’s α
Attitude Towards Using	ATT1	0.926	3.634	1.035	4.124	0.862	0.962	0.947
	ATT2	0.943	3.734	1.052	5.233			
	ATT3	0.915	3.493	1.145	3.614			
	ATT4	0.931	3.718	1.049	4.345			
Behavioral Intention to Use	BI1	0.832	2.877	1.431	2.439	0.777	0.933	0.905
	BI2	0.91	3.598	1.089	3.209			
	BI3	0.903	3.229	1.19	3.28			
	BI4	0.88	3.644	1.051	2.737			
Course Design	CD1	0.872	3.612	1.016	2.556	0.778	0.933	0.905
	CD2	0.873	3.68	1.034	2.509			
	CD3	0.902	3.714	1.033	3.096			
	CD4	0.88	3.775	0.993	2.56			
Computer Self-Efficacy	CSE1	0.881	3.948	0.953	2.63	0.788	0.937	0.91
	CSE2	0.859	4.093	0.886	2.317			
	CSE3	0.921	3.903	0.975	3.778			
	CSE4	0.889	3.825	0.992	2.972			
Information Quality	IQ1	0.919	3.777	1.014	3.789	0.87	0.964	0.95
	IQ2	0.943	3.797	0.96	5.168			
	IQ3	0.937	3.883	0.958	4.965			
	IQ4	0.93	3.853	0.941	4.421			
Learning Outcomes	LO1	0.87	3.608	1.061	2.791	0.842	0.955	0.937
	LO2	0.938	3.584	1.087	4.544			
	LO3	0.925	3.652	1.09	4.593			
	LO4	0.937	3.66	1.054	5.071			
Perceived Ease of Use	PEU1	0.879	3.942	0.976	2.684	0.765	0.929	0.897
	PEU2	0.848	4.32	0.862	2.202			
	PEU3	0.841	3.859	1.122	2.232			
	PEU4	0.927	4.115	0.941	3.846			
Perceived Playfulness	PP1	0.926	3.535	1.141	5.078	0.852	0.959	0.942
	PP2	0.943	3.565	1.083	5.869			
	PP3	0.913	3.604	1.068	3.609			
	PP4	0.911	3.65	1.074	3.632			
Perceived Usefulness	PU1	0.918	3.889	1.011	3.699	0.834	0.953	0.934
	PU2	0.933	3.757	1.069	4.764			
	PU3	0.925	3.716	1.078	4.337			
	PU4	0.876	4.076	0.955	2.626			
System Quality	SQ1	0.886	4.155	0.871	3.25	0.815	0.946	0.924
	SQ2	0.908	4.087	0.901	3.33			
	SQ3	0.938	4.07	0.903	4.616			
	SQ4	0.878	3.883	1.013	2.8			
User Satisfaction	US1	0.9	3.722	1.086	3.03	0.813	0.946	0.923
	US2	0.875	3.92	0.947	2.552			
	US3	0.916	3.579	1.13	3.868			
	US4	0.916	3.606	1.092	3.783			

On the other hand, Tables 4 and 5 present the Fornell-Larcker criterion and the Heterotrait-Monotrait ratio, respectively. These two statistical tests evaluate the discriminant validity to know the extent to which one construct is distinct from another [18]. For the Fornell-Larcker criterion, the results show that the square root of each construct’s AVE is higher than its correlations with other constructs, thereby confirming discriminant validity [18]. Meanwhile, for the HTMT ratio, the results show that all the HTMT values, except for the values of BI and ATT, US and LO, SQ and PEU, and US and PU, are less than the threshold value of 0.9, which signifies a verified discriminant validity [18].

**Table 4:** Fornell-Larcker criterion.

	ATT	BI	CD	CSE	IQ	LO	PEU	PP	PU	SQ	US
ATT	0.929	-	-	-	-	-	-	-	-	-	-
BI	0.889	0.882	-	-	-	-	-	-	-	-	-
CD	0.718	0.652	0.882	-	-	-	-	-	-	-	-
CSE	0.726	0.639	0.676	0.888	-	-	-	-	-	-	-
IQ	0.72	0.647	0.74	0.735	0.933	-	-	-	-	-	-
LO	0.785	0.762	0.745	0.684	0.753	0.918	-	-	-	-	-
PEU	0.648	0.556	0.659	0.739	0.772	0.646	0.875	-	-	-	-
PP	0.807	0.776	0.73	0.731	0.74	0.828	0.632	0.923	-	-	-
PU	0.772	0.701	0.743	0.744	0.842	0.82	0.77	0.79	0.913	-	-
SQ	0.661	0.553	0.667	0.715	0.769	0.623	0.851	0.639	0.755	0.903	-
US	0.825	0.771	0.748	0.7	0.819	0.865	0.71	0.811	0.861	0.717	0.902

**Table 5:** HTMT ratio.

	ATT	BI	CD	CSE	IQ	LO	PEU	PP	PU	SQ	US
ATT	-	-	-	-	-	-	-	-	-	-	-
BI	0.953	-	-	-	-	-	-	-	-	-	-
CD	0.774	0.712	-	-	-	-	-	-	-	-	-
CSE	0.781	0.691	0.744	-	-	-	-	-	-	-	-
IQ	0.759	0.686	0.796	0.791	-	-	-	-	-	-	-
LO	0.833	0.822	0.805	0.74	0.797	-	-	-	-	-	-
PEU	0.703	0.603	0.729	0.816	0.835	0.703	-	-	-	-	-
PP	0.854	0.835	0.789	0.788	0.782	0.881	0.687	-	-	-	-
PU	0.821	0.751	0.806	0.808	0.894	0.876	0.842	0.842	-	-	-
SQ	0.704	0.589	0.725	0.779	0.818	0.665	0.932	0.682	0.812	-	-
US	0.883	0.835	0.817	0.763	0.874	0.927	0.78	0.87	0.927	0.774	-

### 4.2. Structural model

**Fig 2:**
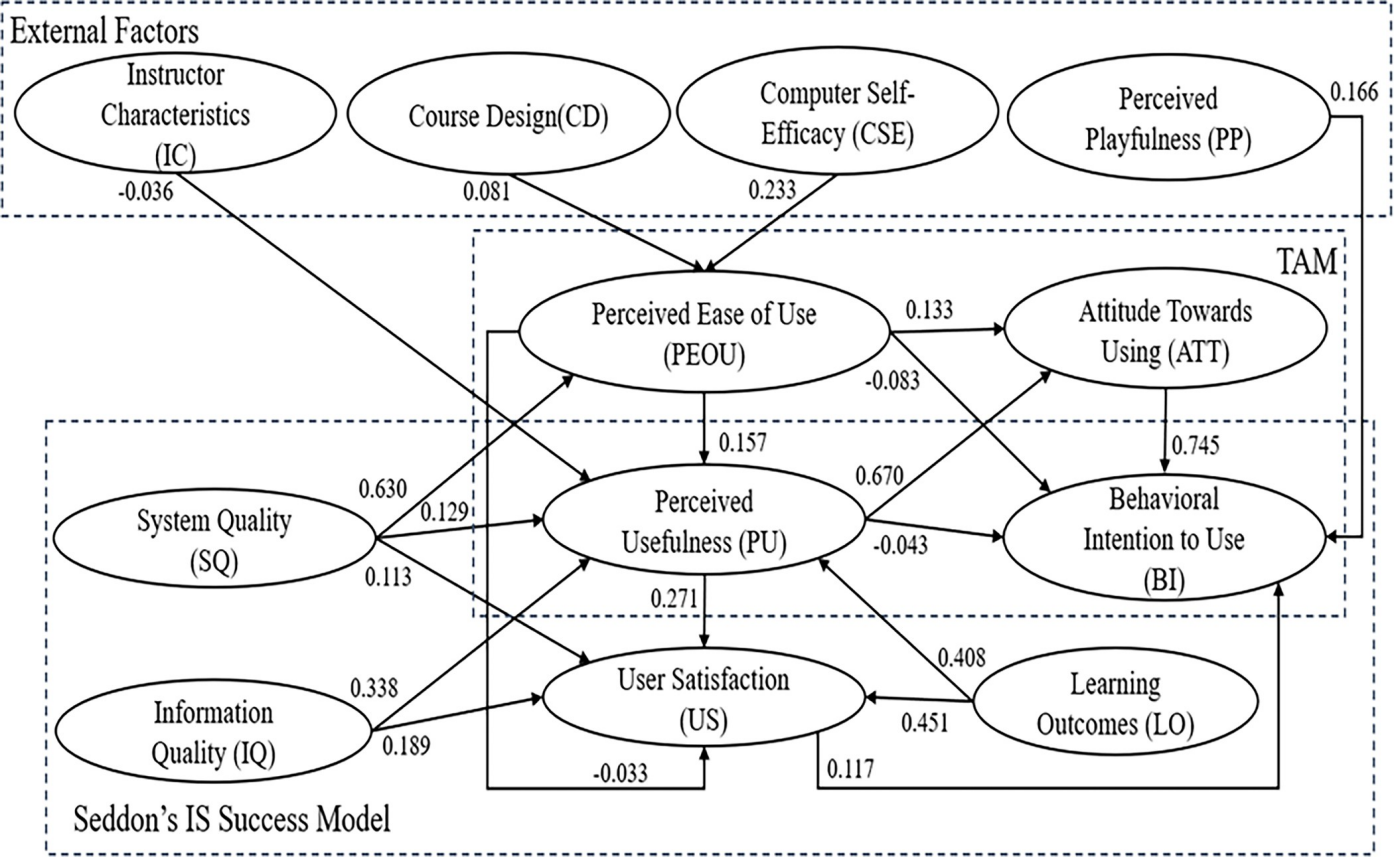
The initial model.

**Fig 3:**
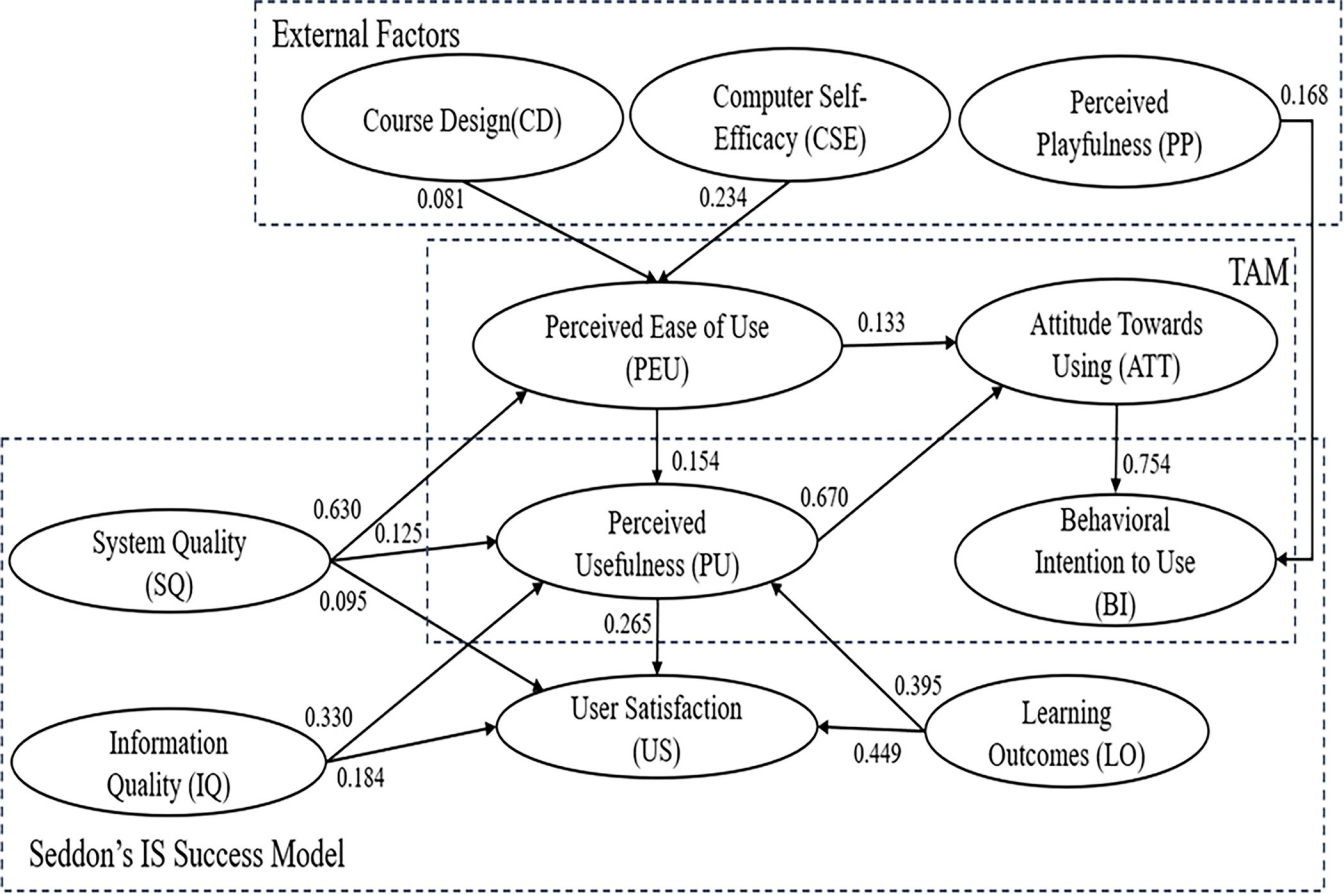
The final model.

After the successful assessment of the measurement model, the structural model was then analyzed to determine the relationship between the proposed variables. The initial model of the study from the bootstrap analysis is shown in Fig 2. Unexpectedly, some paths were not found to be significant: PEU→US, PU→BI, and IC→PU (Table 6). The adequacy of model fit was defined based on statistical tests. The model modification was performed by deleting the latent that is not statistically significant [56]. After removing insignificant paths on the initial model, the resulting model showed that PEU→BI and US→BI were non-significant. Thus, these paths were also removed, and an amended final model was derived. Fig 3 presents the final framework of the study.

**Table 6:** Model modification (p***<0.001).

Relationship		Initial model		Final model		Decision
		β	p-value	β	p-value	
1	SQ → US	0.113	0.025	0.095	0.013	Accepted
2	SQ → PU	0.129	0.008	0.125	0.009	Accepted
3	SQ → PEU	0.63	***	0.63	***	Accepted
4	PEU → PU	0.157	0.002	0.154	0.002	Accepted
5	PEU → BI	-0.083	0.011	-	-	Rejected
6	PEU → US	-0.033	0.545	-	-	Rejected
7	PEU → ATT	0.133	0.005	0.133	0.005	Accepted
8	IQ → PU	0.338	***	0.33	***	Accepted
9	IQ → US	0.189	***	0.184	0.001	Accepted
10	PU → BI	-0.043	0.406	-	-	Rejected
11	PU → US	0.271	***	0.265	***	Accepted
12	PU → ATT	0.67	***	0.67	***	Accepted
13	US → BI	0.117	0.029	-	-	Accepted
14	LO → US	0.451	***	0.449	***	Accepted
15	LO → PU	0.408	***	0.395	***	Accepted
16	IC → PU	-0.036	0.256	-	-	Rejected
17	CD → PEU	0.081	0.047	0.081	0.042	Accepted
18	CSE → PEU	0.233	***	0.234	***	Accepted
19	PP → BI	0.166	0.003	0.168	***	Accepted
20	ATT → BI	0.745	***	0.754	***	Accepted

For the final model fit measures, Table 7 exhibits the results of the Standardized Root Mean Squared Residual (SRMR) and Normed Fit Index (NFI). These are used to evaluate the goodness-of-fit for structural equation models. A lower SRMR value indicates a better fit between the model and the data, with a threshold of 0.08 considered acceptable [57]. Conversely, a higher NFI value signifies a better fit, with a value above 0.6 indicating an acceptable fit [58].

**Table 7:** Model fit measures.

Goodness of fit measures of the SEM	Parameter Estimates		Range	Recommended by
	Initial Model	Final Model		
Standardized Root Mean Square (SRMR)	0.072	0.075	0.00 to 0.08	Hu & Bentler [57]
Normed Fit Index (NFI)	0.857	0.858	0.60 to 1.00	Oke et al. [58]

### 4.3. Measuring the value of *R*^2^

The method for evaluating the model’s predictive capability relies on the proportion of variance. A successful path model requires high *R*^2^ values for key components. As stated by Meet et al. [x], an *R*^2^ value of 0.75 and 0.50 are deemed substantial and moderate, respectively. As shown in Table 8, the *R*^2^ values are substantial. This indicates that the model has a sufficient fit and predictive relevance.

**Table 8:** R square.

	R Square	R Square Adjusted
ATT	0.603	0.601
BI	0.8	0.799
PEU	0.761	0.76
PU	0.815	0.813
US	0.836	0.834

### 4.4. Measuring the value of effect size *F*^2^

The effect size measures the impact on the dependent variable from each of the latent variables. When PLS-SEM is employed, excluding an independent variable helps assess the change in squared correlation values, determining if the excluded variable significantly impacts the dependent variable. In addition, the effect of the predictor variable is high, medium, and low if their corresponding values are 0.35, 0.15, and 0.02, respectively. With this, Table 9 presents the values of the effect size accordingly.

**Table 9:** F square.

	ATT	BI	CD	CSE	IQ	LO	PEU	PP	PU	SQ	US
ATT		0.991									
BI											
CD							0.013				
CSE							0.095				
IQ									0.158		0.049
LO									0.355		0.382
PEU	0.018								0.031		
PP		0.049									
PU	0.46										0.082
SQ							0.711		0.021		0.02
US											

## 5. Discussions and implications

This study combined the extended Technology Acceptance Model, and Seddon’s IS Success Model to evaluate Students’ Intention to an e-learning platform in a Collegiate Aviation Institution in the Philippines during the COVID-19 Pandemic. The interrelationship among latent variables was analyzed using the Structural Equation Modeling approach.

Attitude towards use directly influenced the behavioral Intention to use the e-learning platform (β: 0.754; p<0.001). Analogous to the findings of Salloum et al. [14]. In addition, Teo & Zhou [61] and Vanduhe et al. [37] found that this factor is the most significant predictor of Intention to use. This relationship is highlighted in the TAM by Davis et al. [62]. Also, under Fishbein & Ajzen’s [53] Theory of Reasonable Action, attitudes were expected to influence behavior, and a positive attitude predisposes to "Approach" tendencies.

Perceived playfulness had a significant direct effect on the Intention to use (β: 0.168; p<0.001). Playfulness is reflected as a reward that positively affects the system’s Intention. The students’ perceived enjoyment affects their Intention to use e-learning.

Perceived Usefulness (β: 0.670; p<0.001) and Perceived Ease of Use (β: 0.133; p:0.005) are found to be positive to the attitude, which is similar to previous research studies [14, 37]. The relationship agrees with the TAM by Davis et al. [62]. The higher effect of PU implies that students value how the platform improves their performance and allows them to accomplish tasks more effectively.

Perceived ease of use was a significant predictor of perceived Usefulness (β: 0.154; p:0.002). Similar to previous studies, the more the platform is perceived as easy to use, the more students will consider that the tool would improve their performance and allow them to carry out their tasks more effectively [1, 14, 19, 38].

The course design was found to have a significant impact on perceived ease of use (β: 0.081; p:0.042). Comparable to Lee et al. [3], learners positively regard e-learning if the design of learning content is improved and is suitable to their needs. On the contrary, Lee [63] found that course attributes had no significant effect on PEU. The present research found that students perceived e-learning as easy to use if they were provided with structured content to meet the requirements of the course or subject.

Among the external factors of TAM, computer self-efficacy had a substantial influence on the perceived ease of use (β: 0.234; p<0.001). Individuals’ technology-related knowledge and ability are strongly linked with their assessment of the e-learning system’s complexity [63].

Parallel with the findings of Hsieh and Cho [44], learning outcomes had a significant impact on perceived Usefulness (β: 0.395; p<0.001). Students’ perception of how the e-learning platform increases their knowledge, improves their grades, and allows them to achieve their learning goals positively influences Usefulness.

System quality (β: 0.125; p:0.009) and information quality (β: 0.330; p<0.001) have a significant positive effect on students’ perceived usefulness. Users value their interaction with the system and its functions. Nevertheless, more importance is placed on the relevance of the content to their course or subject. Contents accessed by students should be relevant in achieving the goals of outcomes-based education.

Information quality (β: 0.184; p:0.001) and system quality (β: 0.095; p:0.013) positively influenced user satisfaction. IQ and SQ represent the quality of an information system [24]. These two factors are crucial in successfully designing e-learning systems [41]. The features of e-learning facilitate continuous motivation to utilize online learning [45]. Institutions must continuously look at how they deliver their curriculum to students to improve satisfaction and learning experience.

Learning outcomes positively affected user satisfaction (β: 0.449; p<0.001). After using the e-learning platform, students’ perceived learning outcomes, which include how it improves their grades, increases knowledge and improves their learning process, strongly affect satisfaction. The overall effect after usage is captured in perceptions such as performance outcomes [24, 44].

Similar to the findings of Rai et al. [25] and Hsieh and Cho [44], perceived Usefulness had a positive influence on user satisfaction (β: 0.265; p<0.001). Since they perceived that using the e-learning tool would enhance their performance, its benefits will be satisfactory.

System quality positively influenced students’ perception of the ease of use (β: 0.630; p<0.001). System quality pertains to the ease of navigation, availability, interaction, attractive features, and presentation [41]. Collegiate aviation institutions and developers of an e-learning platform should prioritize SQ in terms of modifications or enhancements made to the platform.

Contrary to expectations, user satisfaction did not significantly affect behavioral Intention. Satisfaction may not have influenced their Intention to use due to the non-volitional setting.

Perceived Usefulness and ease of use were not found to significantly impact behavioral Intention as opposed to previous research [14, 33, 38, 40]. However, the effects of PU and PEU on behavioral Intention were mediated by attitude toward using, which strongly affects behavioral Intention to use. Venkatesh and Davis [64] stated that in some research, the direct effect of perceived ease of use on behavioral Intention had been observed to lessen over time. However, there is no sufficient theoretical rationale to explain changes over time in the PU-BI and PEU-BI relationship. A possible explanation could be that the mandatory usage of e-learning caused the non-significance of PU and PEU on BI. In addition, online learning was implemented for two years and six months, and students have been actively using the e-learning platform. Today, schools still limit face-to-face classes, including Higher Educational Institutions [65, 66].

Dissimilar to Seddon’s IS Model [24] and Sun et al. [45], ease of use had no significant effect on user satisfaction. A probable reason could be the similarity of SQ and Ease of Use. In the study of Hsieh and Cho [44], system quality was measured as ease of use. However, Prasetyo et al. [33] argued that system quality and ease of use should be separated, and PEU should be focused on the utilization of the system.

Contrary to set expectations, instructor characteristics were not found to influence perceived Usefulness. Since not every Instructor has live sessions in teaching online, the learning process may lean towards how students are motivated to study independently [67, 68]. Another probable reason is that the features of the e-learning platform have a more significant role in how students can meet their learning goals. However, it is worthy of attention that the role of instructors is crucial, especially in preparing the course content, which is seen in the significance of IQ and SQ constructs.

### 5.1. Theoretical contributions

The paper represents a significant step towards developing a theoretical understanding of integrating the TAM and Seddon’s IS model in e-learning. Relevant insights are provided for educators, software developers, and policy-making institutions on which constructs employ the most substantial influence on student satisfaction and behavioral Intention. The framework would be valuable for enhancing e-learning infrastructure, especially in collegiate aviation.

### 5.2. Practical contributions

The results of the study exhibit insights that can improve the delivery of subjects and enhance students’ learning experience. The short preparation time of institutions due to the COVID-19 Pandemic may not have allowed sufficient information to meet the student’s goals in the new delivery method. Thus, an extensive understanding of e-learning adoption can aid educators and policymakers in developing best practices to facilitate the delivery of quality education. For developers, the study emphasizes the importance of the interaction of students with the e-learning platform. Maintaining and developing ease of use, user satisfaction, and Usefulness aids students in achieving their desired learning outcomes. For future researchers, this presents a considerable contribution to developing integrated TAM and IS success models in evaluating e-learning acceptance since distance learning in aviation institutions is most likely for part-time students [4].

### 5.3. Limitations and future research

Despite the significant findings of the current study, several limitations must be addressed and future research directions must be discussed. This paper is limited to the technology acceptance and Seddon’s IS model. As stated by Hsieh and Cho [44], constructs should not be the same over time to describe the IS Success model’s dynamics fully. A longitudinal study could also be performed to study the changes in perceptions and preferences over time [14]. Future researchers can utilize different theories in psychology. Furthermore, future studies may compare institutions and platforms, which may be a good topic. Lastly, future studies may be employed to evaluate e-learning in aviation institutions in other countries.

## 6. Conclusion

In higher educational institutions in the Philippines, e-learning has been the primary medium of instruction as face-to-face classes are still limited. The Technology Acceptance Model and Seddon’s IS Model were integrated to explain the Intention of an e-learning platform in a collegiate aviation institution in the Philippines amidst the ongoing worldwide health crisis. Five hundred three students answered the questionnaire with 48 questions representing 12 latent variables. Structural equation modeling was utilized to analyze the interrelationship of the latent constructs [69].

Based on the Structural Equation Model, attitude towards use (ATT) was a strong determinant of behavioral Intention (BI). The result was succeeded by perceived playfulness (PP). Learning outcomes (LO) had the highest impact on perceived Usefulness (PU), succeeded by information quality (IQ), perceived ease of use (PEU), and system quality (SQ). LO was also observed to have the highest effect on students’ user satisfaction (US), succeeded by PU, IQ, and SQ. PU had a more significant influence on ATT than PEU. SQ had the most influence on PEU, followed by computer self-efficacy (CSE) and course design (CD). Interestingly, PEU was not found to affect BI and US. PU and US were observed to have no significant effect on BI. Lastly, the instructor characteristics’ (IC) influence on PU was not found to be significant.
